# Blood-Brain Barrier Breakdown after Embolic Stroke in Rats Occurs without Ultrastructural Evidence for Disrupting Tight Junctions

**DOI:** 10.1371/journal.pone.0056419

**Published:** 2013-02-26

**Authors:** Martin Krueger, Wolfgang Härtig, Andreas Reichenbach, Ingo Bechmann, Dominik Michalski

**Affiliations:** 1 Institute of Anatomy, University of Leipzig, Leipzig, Germany; 2 Paul Flechsig Institute of Brain Research, University of Leipzig, Leipzig, Germany; 3 Department of Neurology, University of Leipzig, Leipzig, Germany; Institute of Neurology (Edinger-Institute), Germany

## Abstract

The term blood-brain barrier (BBB) relates to the ability of cerebral vessels to hold back hydrophilic and large molecules from entering the brain, thereby crucially contributing to brain homeostasis. In fact, experimental opening of endothelial tight junctions causes a breakdown of the BBB evidenced as for instance by albumin leakage. This and similar observations led to the conclusion that BBB breakdown is predominantly mediated by damage to tight junction complexes, but evidentiary ultrastructural data are rare. Since functional deficits of the BBB contribute to an increased risk of hemorrhagic transformation and brain edema after stroke, which both critically impact on the clinical outcome, we studied the mechanism of BBB breakdown using an embolic model of focal cerebral ischemia in Wistar rats to closely mimic the essential human pathophysiology. Ischemia-induced BBB breakdown was detected using intravenous injection of FITC-albumin and tight junctions in areas of FITC-albumin extravasation were subsequently studied using fluorescence and electron microscopy. Against our expectation, 25 hours after ischemia induction the morphology of tight junction complexes (identified ultrastructurally and using antibodies against the transcellular proteins occludin and claudin-5) appeared to be regularly maintained in regions where FITC-albumin massively leaked into the neuropil. Furthermore, occludin signals along pan-laminin-labeled vessels in the affected hemisphere equaled the non-affected contralateral side (ratio: 0.966 *vs.* 0.963; P = 0.500). Additional ultrastructural analyses at 5 and 25 h after ischemia induction clearly indicated FITC-albumin extravasation around vessels with intact tight junctions, while the endothelium exhibited enhanced transendothelial vesicle trafficking and signs of degeneration. Thus, BBB breakdown and leakage of FITC-albumin cannot be correlated with staining patterns for common tight junction proteins alone. Understanding the mechanisms causing functional endothelial alterations and endothelial damage is likely to provide novel protective targets in stroke.

## Introduction

Ischemic stroke still represents one of the leading causes of death worldwide [1]. On the cellular level, the ischemia-induced breakdown of the blood-brain barrier (BBB), which is known to contribute to an increased risk of hemorrhagic transformation and brain edema with perilous outcome, was often linked to a putative disruption of tight junctions in the endothelial layer of cerebral vessels [2]. However, this putative mechanism of breakdown has never been convincingly proven by ultrastructural analyses in conjunction with physiological conditions.

Originally, the observation that cerebral vessels hold back hydrophilic molecules from entering the brain was first described by Paul Ehrlich in 1885 [3]. In 1900, Max Lewandowski drew the revolutionary conclusion that the morphological correlate of this barrier function indeed must be the capillary wall [4], which was further specified by the groundbreaking paper from Reese and Karnovsky in 1967, who linked this property to belts of endothelial tight junctions in cerebral capillaries [5]. These complexes consist of three major transmembrane protein families comprising occludin, claudins and junction associated proteins (JAMs), as well as several cytoplasmic proteins including the zonula occludens (ZO) protein family (ZO-1, ZO-2 and ZO-3), providing the link to the actin cytoskeleton [2], [6], [7]. Whereas this general composition is also known for epithelial tight junctions of peripheral organs, some proteins were found to be specific for brain endothelial cells, such as cingulin, claudin-3 and -5 [8], [9].

Under physiological conditions cerebral endothelial cells ensure the outstanding cerebral barrier function, which is maintained by the critical interaction of pericytes, astrocytes, neurons and the extracellular matrix as parts of the neurovascular unit (NVU) [7]. In this context, astrocytes were originally thought to induce BBB characteristics in cerebral endothelial cells [10]–[13], but also pericytes were shown to enhance their barrier function [14], [15]. In the concert of cells constituting the NVU the establishment and maintenance of the BBB represents a permanently regulated process, which begins in early development and lasts throughout ageing [16]–[18]. The criticial impact of proper endothelial function is exemplarily demonstrated by experimental disruption of endothelial tight junctions, which resulted in generation of epileptiform activity by failure of cellular homeostasis in the NVU [19].

During ischemic stroke, alterations in cerebral blood flow by acute vessel occlusion result in energy failure of the NVU, thereby causing mitochondrial damage, release of toxic oxygen species and intracellular edema, which critically impact on the clinical outcome [20]–[24]. Furthermore, upregulation of the matrix metalloproteinases (MMP)-2 and -9, as well as of the vascular endothelial growth factor (VEGF) are closely associated with BBB disruption [25], [26]. In the past, this event was predominantly linked to tight junction failure, consequently leading to an extravasation of blood-borne molecules along the paracellular route [2], [27], [28]. Indeed, a variety of studies reported on alterations in the expression or localization of critical tight junction proteins, such as occludin, claudin-5 and ZO-1 in diverse models of *in vitro* hypoxia as well as of global and focal cerebral ischemia in rodents (e.g., [26]–[30]). Despite these efforts, a conclusive concept of BBB breakdown during ischemia is currently lacking. The present situation is thus characterized by a discrepancy between the well known clinical impact of the BBB integrity during ischemic stroke and its poorly understood mechanisms, which might impede further therapeutic interventions [31], [32]. Concerning translational issues, the used tracers for visualizing BBB leakage were recently accounted for the risk to inherit a methodological bias due to own intrinsic effects. For instance, Evans blue as probably the most frequently applied agent [25], [33]–[37] was found to inhibit the uptake of glutamate [38], [39] as one of the key mediators during the ischemic cascade [20]. Furthermore, in reference to failures of more than 1,000 neuroprotective approaches in stroke [40], a critical discussion came up addressing the comparability of routinely used rodent models due to their preponderant artificial techniques for vessel occlusion [41], [42].

The present study aimed at the ultrastructural analysis of tight junction integrity after embolic middle cerebral artery occlusion (eMCAO) in rats, which is currently believed to best mimic the human pathophysiology [43]. To investigate the fate of endothelial tight junctions in regions of ischemia-related BBB breakdown, we focused on areas exhibiting fluorescein isothiocyanate (FITC)-labeled albumin (FITC-albumin) extravasation. Multiple fluorescence labeling was utilized to localize endothelial tight junction proteins, while the ultrastructural integrity of tight junctions and routes for tracer extravasation were addressed by electron microscopy.

## Materials and Methods

### Study setup and surgical procedure

Experiments involving animals were performed according to the European Communities Council Directive (86/609/EEC) after protocol approval by local authorities (Landesdirektion Leipzig, Germany, reference numbers TVV 02/09 and 34/11). Generally, efforts were made to minimize the total number of animals and suffering of animals, which were housed in a temperature (21–22°C) and humidity (45–60%) controlled room with 12 hours of light/dark cycle and free access to food and water.

Overall 13 male Wistar rats weighing 322±33 g, bred by Charles River (Sulzfeld, Germany), were subjected to right-sided eMCAO as originally described by Zhang and coworkers [44] with some minor modifications. Briefly, after surgical exposure of right-sided cervical arteries, a polyethylene (PE) tube with tapered end and a maximum outside diameter of 0.4 mm was introduced into the external carotid artery and moved forward through the internal carotid artery up to the origin of the middle cerebral artery (about 1.6 cm from carotid bifurcation). A blood clot (length: 5±0.5 cm) – prepared from blood collected in a PE tube at the previous day and allowed to clot on a warming pad (37°C) for 2 hours followed by overnight storage at 4°C – was injected with about 40 µl of saline. After catheter removal and ligation of the external carotid artery stump, the wound was closed. PE tubes were further inserted into the femoral vein to allow future administration of FITC-albumin and femoral artery to control vital parameters during surgery. Generally, the surgical procedure was done in anesthesia using 2.0 to 2.5% isoflurane (Isofluran Baxter, Baxter, Unterschleißheim, Germany; mixture: 70% N_2_O/30% O_2_; vaporisator: VIP 3000, Matrix, New York, USA). To avoid anesthesia-related cooling during surgery, the body temperature was adjusted to 37.0°C by a thermostatically controlled warming pad with rectal probe (Fine Science Tools, Heidelberg, Germany). After surgery, animals spent time on a commercial warming pad (37.0°C) until recovery from anesthesia. Sufficient eMCAO induction was ensured by functional assessment using Menzies score (basically ranging from 0 ‘no apparent deficit’ to 4 ‘spontaneous contralateral circling’; [45]), whereas animals had to show a functional relevant deficit during observation period as indicated by a score of at least 2. Post-surgical pain control was ensured by metamizol- or paracetamol-enriched (Novaminsulfon-ratiopharm, Paracetamol-ratiopharm, ratiopharm, Ulm, Germany) drinking water.

Four or 24 hours after ischemia onset, FITC-albumin (20 mg/1 ml saline; Sigma, Taufkirchen, Germany) was intravenously administered to allow fluorescence-based localization of BBB breakdown, indicated by FITC-albumin leakage [46]. After a circulation period of usually 1 hour, animals were deeply anesthetized using CO_2_ and transcardially perfused with either 200 ml saline (n = 5, for immunohistological analyses by fluorescence microscopy at 25 hours), or 200 ml saline followed by 200 ml of a phosphate-buffered fixative containing 4% paraformaldehyde and 0.5% glutaraldehyde (n = 4 each, for ultrastructural analyses by electron microscopy at 5 and 25 hours). After extraction of the brains the samples for fluorescence microscopy were immediately snap frozen in isopentane on dry ice.

### Fluorescence microscopy and quantification of tight junction integrity

For fluorescence microscopy, the brains were coronally cut in 10 µm sections on a cryotome (Leica Microsystems, Wetzlar, Germany). Before starting the staining procedure, the samples were post-fixed with Zinc-formalin for 5 minutes at room temperature followed by several rinses with 0.1 M phosphate-buffered saline, pH 7.4 (PBS). Unspecific binding of the antibody was inhibited by a blocking step applying 5% of normal goat serum (NGS) and 0.3% of Triton X-100 in PBS for 20 min. The primary antibodies mouse anti-occludin (Antibodies-online, Aachen, Germany; 1∶200), rabbit anti-claudin-5 (Abcam, Cambridge,UK; 1∶200), rabbit anti-laminin (Dako, Hamburg, Germany; 1∶200) and guinea pig anti-GFAP (Synaptic Systems, Göttingen, Germany; 1∶200) were allowed to incubate over night at 4°C in PBS containing 0.5% NGS. After several washing steps, the sections were reacted with highly purified fluorochromated antibodies (Dianova, Hamburg, Germany; diluted in PBS containing 0.5% NGS) specifically recognizing IgGs from the host species of primary antibodies for 2 hours. Subsequently, sections were washed several times in PBS and coverslipped with fluorescence mounting medium (Dako, Hamburg, Germany). Omitting the primary antibodies in control experiments resulted in the expected absence of staining. The specimens were analyzed with an Olympus BX51 fluorescence microscope (Olympus, Hamburg, Germany) equipped with a digital camera.

For quantification of tight junction integrity, the ratio of tight junction-positive vessels with respect to the total number of vessels was captured in the area of FITC-albumin leakage, as indicator of BBB breakdown, and compared to the corresponding region on the contralateral hemisphere. Therefore, we applied double immunofluorescence staining for occludin as marker for endothelial tight junctions and pan-laminin as a general marker for cerebral vessels [47]. To ensure exclusive analysis of ischemia-affected areas on the ipsilateral hemisphere depicting BBB breakdown, we focused on regions showing a clear FITC-albumin extravasation into the neuropil. Low power (10× objective) magnification was used to count the number of occludin-positive vessels in relation to the total number of laminin-immunopositive vessels in usually 8 non-overlapping optic fields per animal and hemisphere, resulting in an amount of about 600 to 1,000 vessels per hemisphere. After calculating the ratio per optic field, a mean of ratios was build per animal and hemisphere. Finally, the global mean of the ipsilateral hemisphere was compared to the contralateral hemisphere, originating from n = 5 animals for each.

### Ultrastructural analysis

For electron microscopy the brains were consecutively cut in 60 µm sections on a vibratome (Leica Microsystems, Wetzlar, Germany) in cooled PBS. The immunohistochemical conversion of FITC into an electron-dense reaction product was performed according to our previous study [45]: After several washing steps in 0.1 M Tris-buffered saline, pH 7.4 (TBS), the sections were blocked with TBS containing 2% bovine serum albumin (TBS-BSA) for 30 min and then incubated with peroxidase-conjugated anti-fluorescein IgG (Dianova, Hamburg, Germany; 1∶2000 = 0.5 µg/ml) in TBS-BSA for 2 hours. Next, the tissue was rinsed with TBS twice and with the substrate buffer (0.05 M Tris, pH 7.6) followed by staining with diaminobenzidine (DAB) as chromogen.

After several washing steps in PBS the sections were stained with 0.5% osmium tetroxide in PBS for 30 min. After 5 washing steps the sections were dehydrated using 30%, 50% and 70% of ethanol. Subsequently, the tissue was incubated with 1% uranyl acetate in 70% ethanol for 1 hour. Following further dehydration using 80%, 90%, 96%, 100% ethanol and finally propylene oxide the sections were incubated in Durcupan (Sigma Aldrich, Steinheim, Germany) and embedded between coated microscope slides and cover slips followed by polymerization at 56°C for 48 hours. After microscopical identification of areas exhibiting FITC-albumin leakage demarked by DAB staining the cover slips were removed and the respective areas were transferred on blocks of resin followed by another step of polymerization at 56°C for 48 hours. Afterwards, the blocks of resin were trimmed down to the areas intended for ultrastructural analysis which were relocated on semi-thin sections and then ultra-thin sections of 60 nm thickness were prepared on an ultramicrotome (Leica Microsystems, Wetzlar, Germany). Finally, sections were transferred on formvar-coated grids and stained with lead citrate for 6 min. Ultrastructural analysis was performed using a Zeiss EM900 transmission electron microscope and a Zeiss SIGMA electron microscope equipped with a STEM detector, respectively (Zeiss NTS, Oberkochen, Germany).

### Statistical analysis

The obtained data on tight junction integrity were processed with IBM SPSS Statistics 20 (IBM Corp., New York, USA). Thereby, the Wilcoxon test was utilized to check for statistical significance between means of ratios. In addition, results were further stressed by adding the Monte Carlo simulation with 10,000 samples and a confidence interval of 99%. Generally, a *P*<0.05 was considered as statistical significant.

## Results

### Identification and characterization of areas showing BBB breakdown 25 hours after ischemia onset

**Figure 1:**
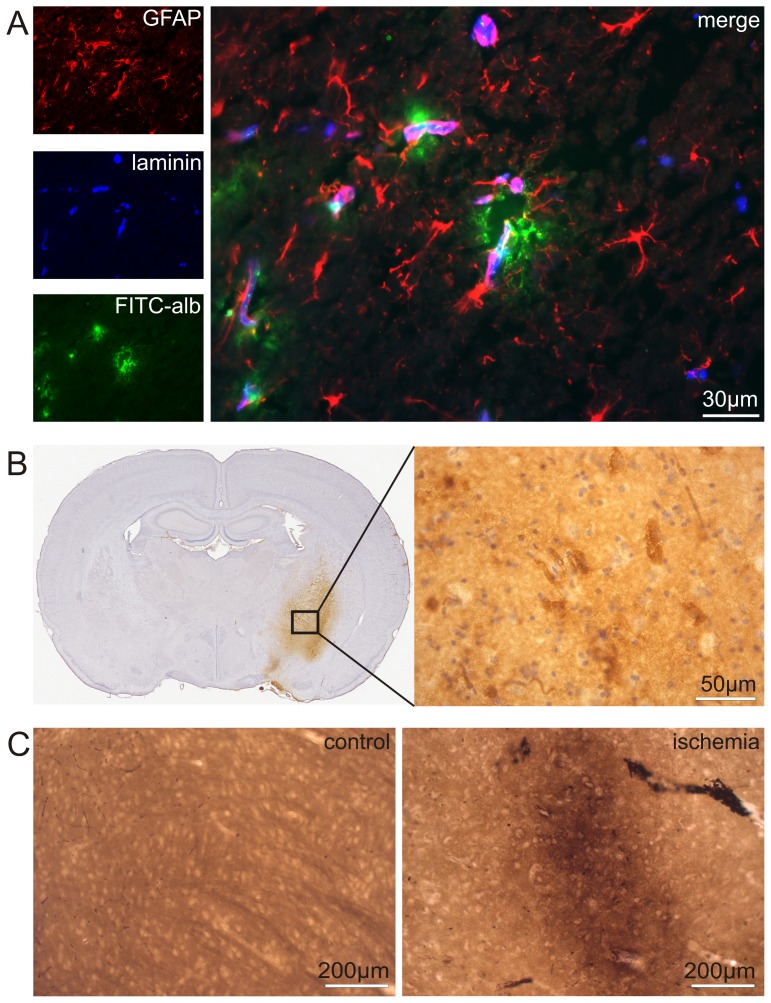
Detection of FITC-albumin leakage following experimental ischemic stroke. (A) Double fluorescence labeling of laminin (color-coded in blue) and GFAP (red) in combination with applied FITC-albumin (green) reveals areas of ischemia-related BBB breakdown. By application of both, an antibody detecting astrocytes (GFAP, red) and an antibody for pan-laminin (blue) which visualizes vascular as well as glial basement membranes [47] the observed leakage can clearly be demonstrated to reach the brain parenchyma proper. Therefore, FITC-albumin is detectable within all the three compartments of the neurovascular unit, represented by the vascular wall (1^st^ compartment), the perivascular space (2^nd^ compartment) and the adjacent neuropil (3^rd^ compartment), delineated by astocytic endfeet (red) forming the glia limitans [54]. (B) To prove specificity of the applied reagent used for conversion of extravasated FITC-albumin into a permanent labeling by DAB for ultrastructural analysis, we exemplarily performed control stainings on vibratome sections, which were not further processed for electron microscopy. The low magnification of a coronary section clearly demonstrates the specificity of the applied reagent and its reaction product (DAB, brown). These sections were counterstained with hemalaun (blue). Areas of FITC-albumin leakage are clearly confined to the striatum. Higher magnification reveals a general leakage of FITC-albumin into the neuropil (brown background). In perivascular and juxtavascular areas the DAB staining appears to be more intense. (C) To confine ultrastructural analysis to areas with BBB breakdown, we identified areas of FITC-albumin extravasation after embedding in resin on coated microscope slides. These areas were selectively processed for ultrastructural analysis by electron microscopy. In corresponding control areas no FITC-albumin extravasation was observed. Please note, the general brown tissue background is a consequence of the embedding procedure using osmium tetroxide and uranyl acetate, which clearly can be distinguished from DAB staining as indicated on the left.

According to our previous study [46], the applied model of eMCAO resulted in alterations of the vascular architecture with a clear extravasation of FITC-albumin in respective areas, primarily located in the right striatum. In contrast, BBB breakdown was not observed on the contralateral hemisphere as the respective areas here were devoid of FITC-albumin leakage. To distinguish leakage of the tracer into the vascular wall or adjacent perivascular spaces from leakage into the neuropil, we applied double fluorescence labeling of vascular basement membranes by combined immunostaining for laminin and astroglial GFAP (Fig. 1A).

For ultrastructural analysis and identification of areas showing FITC-albumin leakage, we made use of a peroxidase-coupled anti-fluorescein IgG and DAB, which served as permanent and electron dense chromogen. To prove the specificity of this technique on glutaraldehyde-treated sections for electron microscopy, control stainings were performed on consecutive vibratome sections. The specificity of the staining was proven by restriction of the DAB signal to the right striatum affected by eMCAO and the absence of any labeling on the contralateral hemisphere (Fig. 1B). To precisely focus further ultrastructural analysis to areas exhibiting extravasation of the tracer, respective areas were identified by light microscopy after embedding of the tissue into resin (Fig. 1C). Importantly, this protocol allowed confinement of ultrastructural analysis to regions of BBB breakdown and their corresponding control areas.

### Expression of critical tight junction proteins in areas of FITC-albumin extravasation

**Figure 2:**
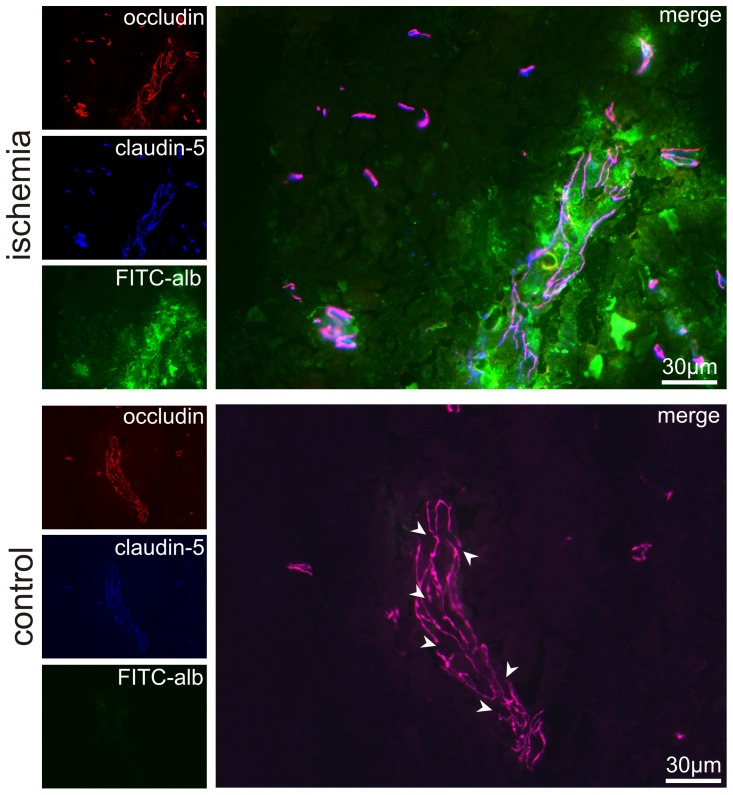
Expression of claudin-5 and occludin in areas of FITC-albumin extravasation. Double fluorescence labeling of claudin-5 (blue) and occludin (red), both being transmembrane proteins critical for tight junction formation, demonstrates extravasation of FITC-albumin (green) in the vicinity of vessels expressing both markers. Please also note the presence of discontinuities in the staining pattern of control vessels with an ‘intact’ endothelial barrier (arrow heads).

To further assess whether belts of tight junctions are altered in areas of FITC-albumin leakage critical tight junction constituents were analyzed by double fluorescence labeling. In contrast to our own expectations, visualization of the essential transmembrane tight junction constituents occludin and claudin-5 revealed presence and regular morphology of both antigens in control areas as well as in areas of BBB breakdown (Fig. 2). Presence of established and still continuous belts of tight junctions in vessels showing bright signals of FITC-albumin in the perivascular and juxtavascular area suggests that extravasation of FITC-albumin does not necessarily depend on changes of staining patterns reflecting a decomposition of endothelial tight junctions in the applied model of eMCAO in rats.

### No difference in the distribution of occludin-positive vessels in areas of FITC-albumin leakage compared to control areas

**Figure 3:**
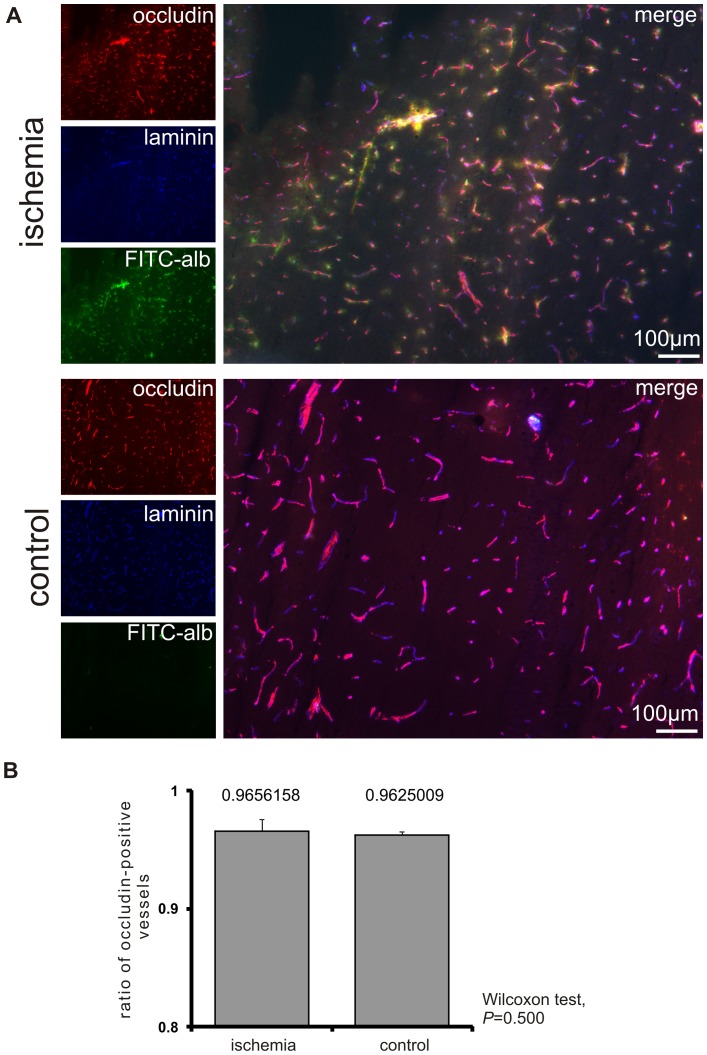
Ratio of occludin-positive vessels in areas of FITC-albumin leakage. (A) Quantitative analysis of differences in the expression of occludin in areas of FITC-albumin extravasation and their corresponding control areas was performed using low power (10× objective) magnification. Here, laminin-immunolabeling revealed the total number of vessels, whereas occludin-immunoreactivity visualized a critical tight junction constituent. The ratio of occludin-positive vessels to the total number of vessels was determined in 5 animals. (B) The ratio of occludin-positive vessels to the total number of vessels did not differ significantly in areas of FITC-albumin leakage and their corresponding control areas. Bars represent means and added lines indicate standard errors.

To investigate the question of whether or not there is a statistical significant difference in the expression of critical tight junction proteins between areas of FITC-albumin extravasation and respective contralateral regions, double fluorescence labeling was applied by use of an antibody for laminin as a pan-vessel marker and the critical transmembrane protein occludin to demark tight junction complexes. Using low power (10× objective) magnification localization of the tracer beyond the glial basement membrane ensured precise confinement of analysis to areas showing distinct FITC-albumin leakage into the brain parenchyma. Here, we compared the number of occludin- and laminin-positive vessels in areas of BBB breakdown and their corresponding control areas (Fig. 3A). As shown in Fig. 3B, nearly identical ratios were found for the ischemia-affected (0.966±0.010) and the contralateral hemisphere (0.963±0.003), impressively failing statistical significance (Wilcoxon test, *P* = 0.500; Monte Carlo simulation, *P* = 0.630).

### Ultrastructural evidence for vascular leakage in areas of morphologically intact tight junctions

**Figure 4:**
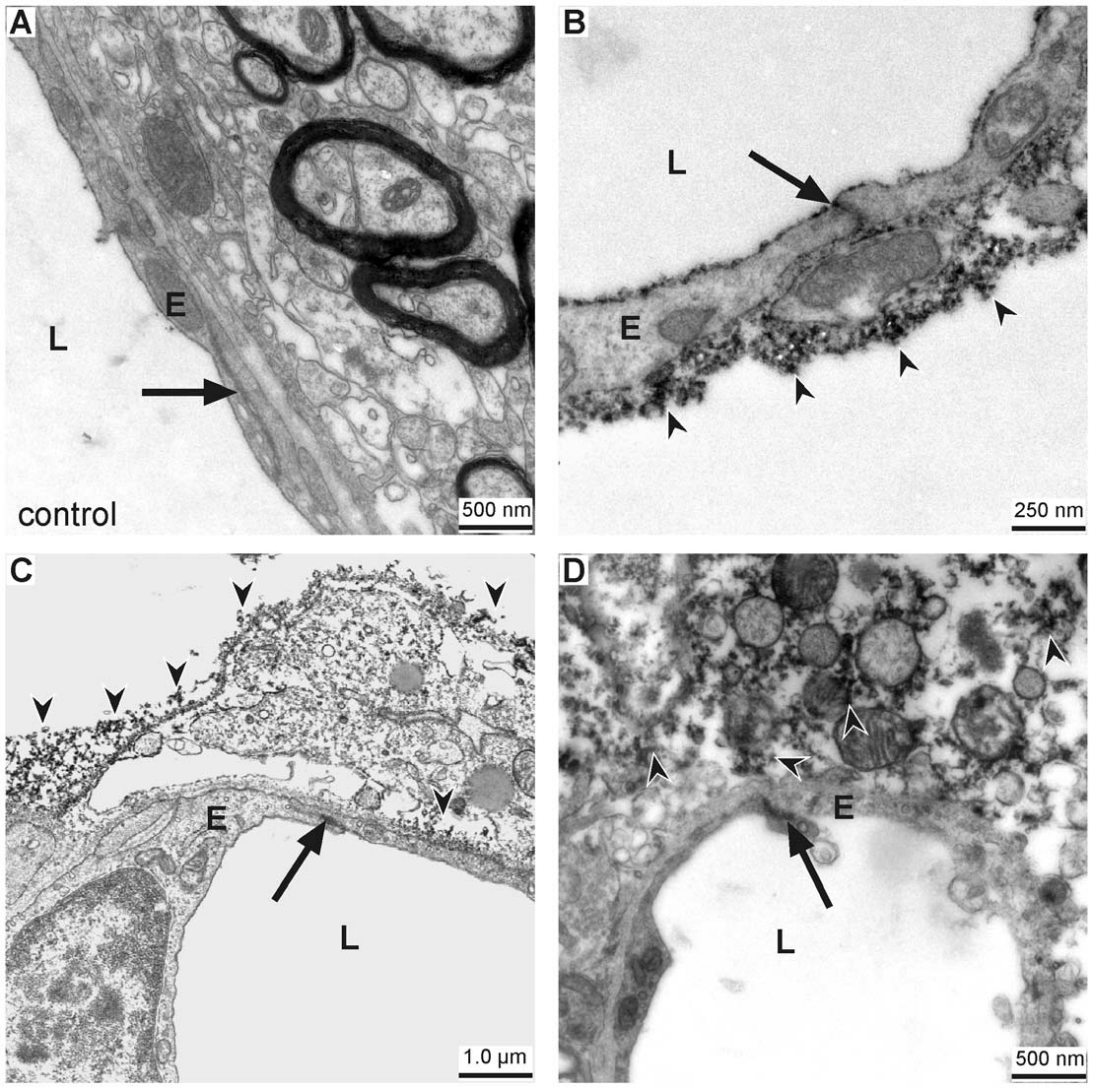
Vascular leakage in areas of ultrastructurally intact tight junctions. (A) Ultrastructural analysis of a control area located on the contralateral hemisphere shows a smooth endothelial layer (E) with an intact tight junction complex (arrow). The surrounding basement membrane is clearly visible and the adjacent neuropil does not show any structural alterations. (B–D) In areas of FITC-albumin extravasation the tight junction complexes (arrows) regularly appear to be established. The adjacent neuropil often displays cellular edema and cellular debris. Extravasated FITC-albumin and its product of conversion (black DAB grains, arrow heads) can constantly be found in the adjacent brain parenchyma proper. E = endothelial cells; L = vascular lumen.

Due to apparent limitations of fluorescence microscopy to elucidate minor changes of tight junction complexes, electron microscopy was applied to reveal ultrastructural alterations responsible for vascular leakage. Using flat-embedded vibratome sections stained with DAB to demark the applied tracer, we were able to precisely identify regions of BBB breakdown and their corresponding control areas by initial light microscopy (Fig. 1C). On the contralateral hemisphere, ultrastructural analysis elucidated intact belts of tight junctions within a smooth endothelial layer showing no ultrastructural alterations. The ensheathing basement membrane was found to be continuous and the adjacent neuropil appeared unaffected (Fig. 4A). In areas of FITC-albumin extravasation, the juxtavascular parenchyma often displayed edema of astrocytic endfeet, in conformity with Ito and coworkers [48], and also cellular debris. On the abluminal side of the vascular basement membrane DAB grains regularly indicated extravasation of the applied tracer. However, in these segments of the vascular tree, the tight junctions were still found to be intact, thus sealing the endothelial layer as shown by fluorescence microscopy before (Fig. 4B–D).

### Ultrastructural evidence for transcellullar, not paracellular leakage

**Figure 5:**
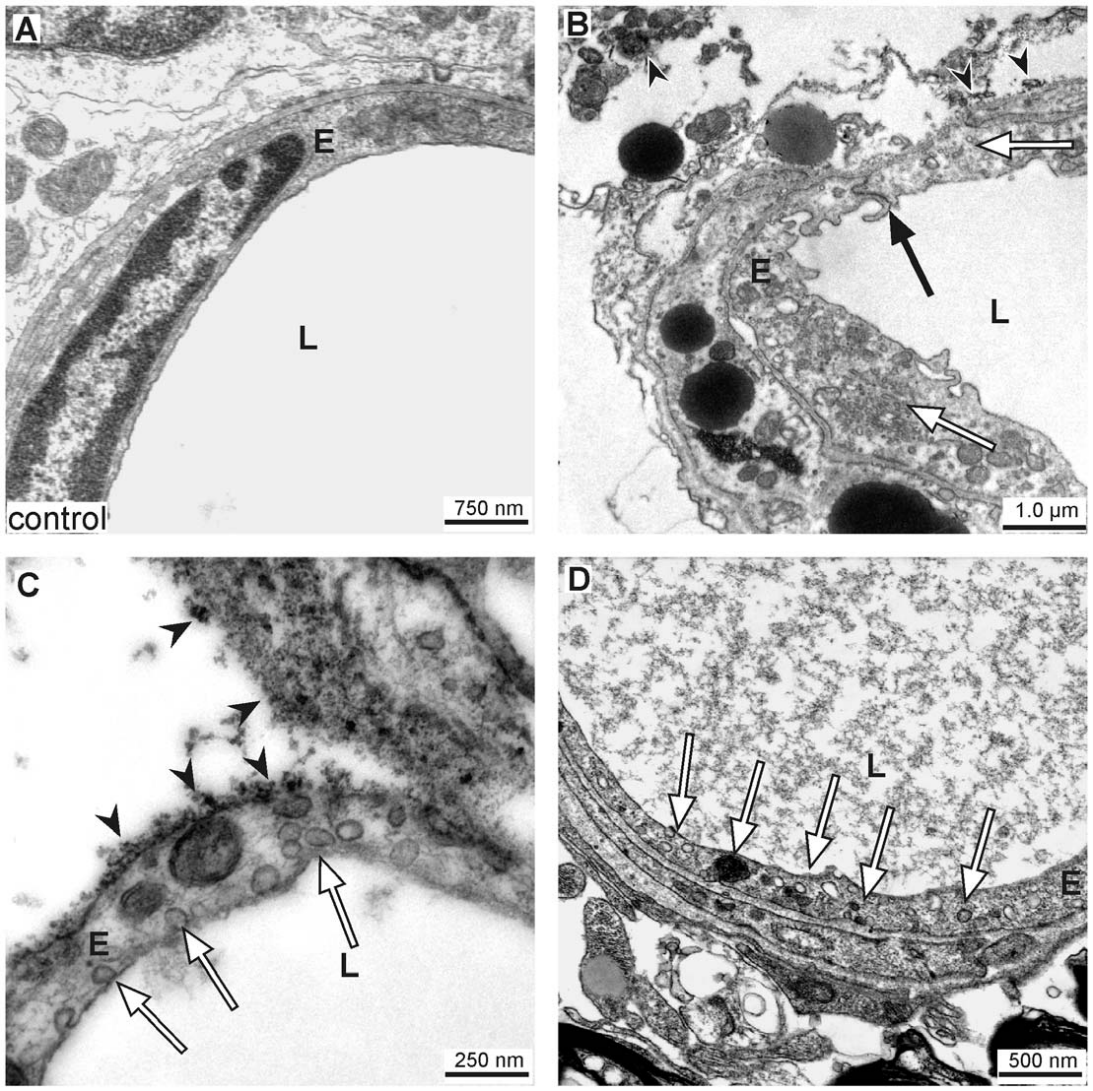
Ultrastructural evidence for transcellular, not paracellular leakage. (A) Vessels in control areas on the contralateral hemisphere do not show signs of a transcellular, vesicle-mediated extravasation of FITC-albumin. (B–D) In contrast to alterations within the belts of tight junctions, ultrastructural examination regularly revealed signs for a transcellular leakage of the tracer. The endothelial cytoplasm exhibits a remarkable increase in vesicle density (white arrows) across the whole vascular circumference. Again, tight junctions (black arrow) are found to be intact. DAB grains are indicated by arrow heads. Control = contralateral hemisphere, E = endothelial cells; L = vascular lumen.

As evidence for leakage through altered endothelial tight junctions was lacking, a more detailed comparison between the vasculature on the control hemisphere and areas of FITC-albumin leakage on the ipsilateral hemisphere was performed to search for signs of transcellular leakage. In control areas, the endothelium appeared not to be affected by the experimental procedure. The nuclei exhibited the typical distribution pattern of euchromatin and heterochromatin, but hardly any transcellular vesicles. Adjacent cells within the neurovascular unit appeared normal and extravasation of the tracer was not observed (Fig. 5A). In contrast, endothelial cells within areas of BBB breakdown often showed a ruffled vascular surface with dramatically increased numbers of cytoplasmic vesicles. As described above, tight junction complexes concomitantly appeared to be intact (Fig. 5B). Around vessels showing the described alterations grains of DAB were regularly observed in adjacent perivascular spaces or within the neuropil. Therefore, we propose that the contribution of leaky belts of tight junctions to the failure of the endothelial BBB characteristics is rather questionable in the model of eMCAO. More likely, it is the result of a failure in suppressing vesicle-mediated leakage through the endothelium itself.

### Evidence for leakage across disintegrated endothelium

**Figure 6:**
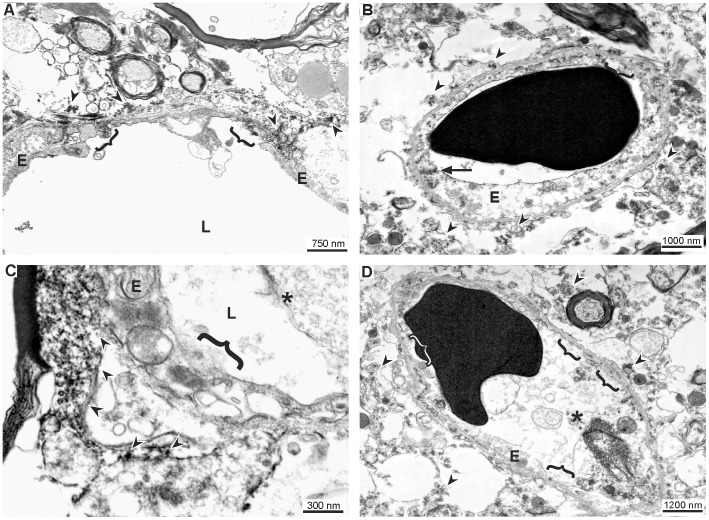
Evidence for leakage across structurally altered endothelium. In addition to the observation of a dramatic increase of the vesicle density, we often observed a disintegration of the whole endothelial layer. In areas exhibiting BBB breakdown the cellular surface of the endothelium was frequently found to be ruffled or discontinuous. Therefore, the vascular wall was often shown to consist of endothelial debris and basement membranes, only (brackets). Thus, in contradiction to a variety of studies our data strongly suggest a transendothelial leakage pattern of affected vessels. DAB grains indicating extravasation of FITC-albumin are demarked by arrow heads. E = endothelial cells; L = vascular lumen; asterisk = cellular debris in the lumen of the vessel; arrow heads = DAB grains; arrow = tight junction.

In addition to the finding of an enhanced vesicle transport through the endothelial layer, a disintegration of endothelial cells was often observed allowing direct extravasation of FITC-albumin into perivascular spaces and the adjacent neuropil. Here, the luminal plasma membrane frequently appeared to be discontinuous. Thus, the former barrier for blood-borne molecules towards the brain parenchyma proper is often constituted by endothelial debris and remnants of the vascular basement membrane only (Fig. 6).

### Equal ultrastructural conditions in the early stage after ischemia onset

**Figure 7:**
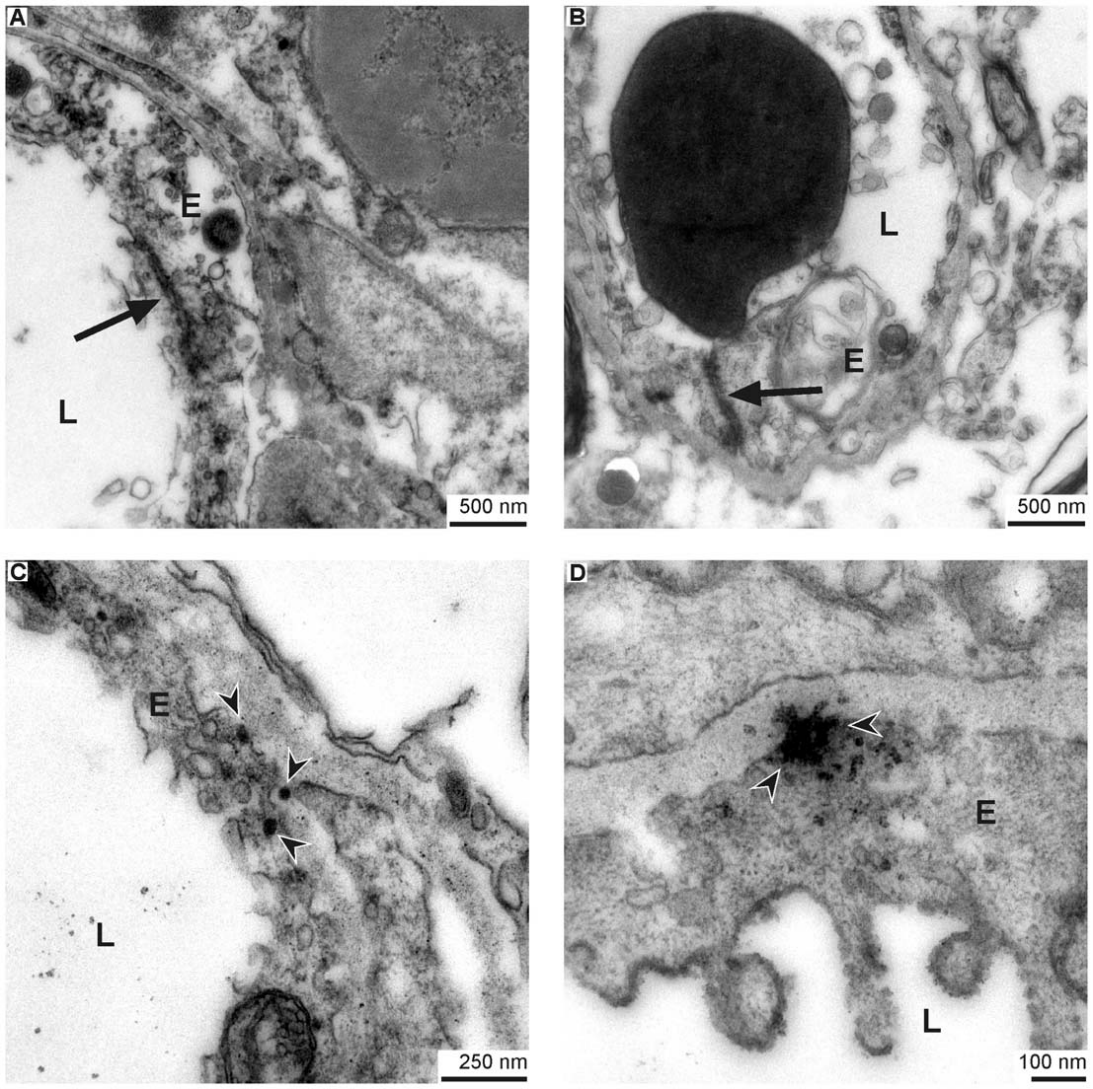
Evidence for FITC-albumin leakage across disintegrated endothelium in the early stroke phase. Finally, the same pattern of extravasation and endothelial damage was found at 5 hours after ischemia induction (A–D), as shown at 25 hours before. While the tight junctions remain, the rest of the endothelial cells may be constituted of debris, only (A and B). Often, the vascular basement membrane is exposed to the vascular lumen (B). Furthermore, electron dense vesicles carrying FITC-albumin can often be observed in the endothelium and the adjacent basement membrane (C) where the content is found to be deployed (D). E = endothelial cells; L = vascular lumen; arrow heads = DAB-filled vesicles; arrow = tight junction.

Finally, we applied electron microscopy at 5 hours after ischemia onset to capture the condition in this early stage of ischemia, and to allow comparison to the previously described 25-hour time point. Thereby, an equal situation was noted, clearly demonstrating regularly established tight junctions in the presence of endothelial damage and FITC-albumin leakage, as well as enhanced vesicle trafficking within the morphologically altered endothelium (Fig. 7).

## Discussion

Despite their enormous clinical relevance, the underlying mechanisms for ischemia-induced brain edema and hemorrhagic transformation at the endothelial level are still poorly understood. Consequently, the regulation of BBB integrity has achieved high priority rating in today's stroke research [31]. In the past, promising concepts of stroke treatment developed by experimental research using different animal models often failed to be transferred into the clinical setting, such as NXY-059 [42], [49]. Since the ways to reproducibly mimic conditions of cerebral ischemia in experimental models are limited, investigators are often forced to make use of artificial and unphysiological ways to decrease blood flow in respective cerebral vessels [41]. Thus, frequently used techniques vary from electrocoagulation or ligation to the internal occlusion of the vascular lumen by inserted filaments [50]. Furthermore, the applied tracers to investigate a putative leakage of the endothelial barrier such as dextrans [51], [52] or the widely utilized Evans blue [25], [34]–[37] generally comprise the risk to produce bias due to immunological side effects or modulation of ischemic key mediators [38], [39], [53], thereby disturbing the physiological environment of the NVU and its constituents. Moreover, investigation of cellular mechanisms and intracellular signaling cascades predominantly requires use of *in vitro* experiments, which are useful to study endothelial function, but not pathophysiological events of the complete NVU including pericytes, astrocytes, neurons and components of the extracellular matrix, which act in concert to maintain the barrier function of the cerebral vasculature [7], [23], [54]–[56].

In order to minimize artificial alterations, we used an embolic model of ischemic stroke in rats, which is believed to best mimic the human pathophysiology [43]. Furthermore, BBB leakage was assessed by injected FITC-labeled albumin which is described to show the same pattern of extravasation as compared to traditional tracers, such as rat IgG with nearly double molecular weight [46]. By limiting the circulation period of FITC-albumin to 60 minutes prior to our observation time points at 5 and 25 hours after ischemia onset, we made sure to precisely capture BBB alterations in this distinct time window. Although ischemia-related BBB breakdown is reported to follow a complex and dynamic pattern, detection of FITC-albumin leakage assured investigation of affected areas in a temporally and locally specific manner. Thereby, FITC-albumin enabled a clear-cut identification of areas with BBB breakdown as known precondition for brain edema and hemorrhage in the clinical situation [57], and the application of double fluorescence labeling for transmembrane tight junction constituents allowed simultaneous analyses of endothelial belts of tight junctions.

Although alterations of tight junctions have been described in several models of hypoxia and stroke before [26], [28], [30], [58], [59], we were not able to correlate areas of FITC-albumin leakage with alterations in the expression pattern of the transmembrane proteins occludin and claudin-5 in this model of focal cerebral ischemia (Fig. 2). This finding was confirmed by a quantitative analysis based on the presence of essential tight junction proteins in areas of FITC-albumin extravasation compared to control regions on the contralateral hemisphere. In detail, immunolabeling of the transmembrane protein occludin demarked endothelial belts of tight junctions and laminin-immunoreactivity served as a vascular basement membrane marker [47], allowing a ratio of occludin-positive vessels to be calculated. Importantly, these ratios did not differ when comparing areas of FITC-albumin leakage and the respective control regions (Fig. 3), indicating not only a lack of qualitative but also of quantitative changes.

To rule out that possible alterations of the endothelial tight junctions remain undetected due to limitations of fluorescence microscopy, electron microscopy was applied to analyze ultrastructural changes within the endothelial layer. As previously described [46], we used the nearly background-free immunohistochemical conversion of FITC-albumin by peroxidase-coupled anti-fluorescein into the electron-dense chromogen DAB to specifically identify areas of BBB breakdown. In fact, by embedding of DAB stained vibratome sections under coated cover glasses, we were able to identify respective areas and the exact location on the contralateral hemisphere before preparation of ultra-thin sections for electron microscopy. Conversion of FITC-albumin into DAB ultrastructurally resulted in a typical granular staining, which was strictly confined to sites of extravasation of the tracer. In line with our findings based on fluorescence microscopy, at both 5 and 25 hours after ischemia onset the tight junction complexes were regularly established although DAB grains clearly indicated extravasation into perivascular spaces or the adjacent neuropil (Fig. 3). These results are clearly contradicting studies describing ultrastructural alterations of tight junctions in diverse models of hypoxia and stroke [28], [60]. However, the traditional link between presence of tight junctions and BBB integrity as well as the variety of valuable data demonstrating altered expression patterns of tight junction proteins in models of hypoxia and stroke [26], [27]–[30], [59] may have constituted a bias affecting interpretation of ultrastructural images. Thus, questionable alterations were addressed as intercellular openings or tight junction loss [28], [60]. On the other hand, previous data also showed molecular alterations of tight junction proteins, mediated as for instance *via* changes in the extra-/intracellular calcium level or phosphorylation of proteins, finally leading to variant functional characteristics [6], [9]. Moreover, concerning molecular formation a recent report identified changes of the extracellular loop 2 of claudin-5 as underlying mechanism for different phenotypes of tight junctions [61]. Taking these findings into consideration, there might be ischemia-related changes in the organization of tight junctions which are not detectable by fluorescence labeling or electron microscopy. This perspective is supported by a previous study showing paracellular tracer leakage but no change in tight junction-associated proteins in an *in vitro* model of endothelial cells [62]. Indeed, the dramatic endothelial damage observed in this study powerfully illustrates that staining patterns of typically used tight junction proteins cannot be correlated with BBB integrity.

However, Reese and Karnovsky who first described endothelial tight junctions as ‘morphological correlate of a BBB’ also considered in the same paper ‘paucity of transcytotic vesicles’ as a mechanism to prevent penetration of hydrophilic molecules into the brain [5]. It is interesting to note that an early study from Ito and coworkers in 1980 investigated the effect of hypertension after cerebral ischemia, and thereby, already suggested an upregulation of the transcellular vesicle transport to result in a transendothelial leakage pattern [63]. Moreover, the concept of a predominant transendothelial route was also mentioned by Dietrich and coworkers in 1987 in a model of photochemically induced cerebral ischemia [64]. However, this reasonable perspective has been widely neglected in the literature.

As we were also not able to demonstrate loss of tight junctions, we further analyzed the areas of tracer extravasation for differences in the distribution of transcytotic and pinocytotic vesicles compared to respective control areas. Indeed, we found a remarkable increase of these vesicles in areas of FITC-albumin leakage while control areas exhibited no or scarcely any vesicles at all (Fig. 5). Furthermore and supporting the hypothesis of a transcellular leakage, we frequently observed a distintegration of the endothelial layer itself, thereby allowing unhindered extravasation of blood-borne molecules at different time points (Fig. 6 and Fig. 7). Given that the alterations revealed in this study were that striking, it is reasonable for us to assume that they may be primarily responsible for the observed BBB breakdown after ischemic stroke. Therefore, our study provides robust evidence that the pathophysiological mechanism leading to hemorrhagic transformation and brain edema in stroke does not necessarily depend on ruptures of endothelial tight junctions in areas of ischemia-related BBB dysfunction. We rather suggest, that in addition to the established paracellular leakage pattern, an endothelial dysfunction leading to an extravasation over the whole surface of affected endothelial cells is likely to critically contribute to the formation of brain edema and its well-known clinical implications. This view can be substantiated by recent data proposing that enhanced vesicle trafficking may be related to an upregulation of caveolin-1 under hypoxic conditions [65]. Thus, effects on the expression of tight junction constituents do not necessarily represent the cause for BBB breakdown but may rather be a consequence of a dysfunctioned endothelial layer which allows transcellular leakage. Although our data need to be complemented by future studies focusing on time points beyond the first day after ischemia onset, since BBB alterations were described to maintain up to days or weeks [66], [67], the present findings might contribute to a better understanding of the NVU under ischemic conditions. In this context, our study may demonstrate that targeting tight junctions alone is inappropriate to address BBB function. Furthermore, the obtained results of a predominantly dysfunctional endothelium may pave the way towards novel neuroprotective strategies by targeting the endothelial layer.

## References

[1] Donnan GA, Fisher M, Macleod M, Davis SM (2008) Stroke. Lancet 371: 1612–1623. 18468545 10.1016/S0140-6736(08)60694-7

[2] Sandoval KE, Witt KA (2008) Blood-brain barrier tight junction permeability and ischemic stroke. Neurobiol Dis 32: 200–219. 18790057 10.1016/j.nbd.2008.08.005

[3] Ehrlich P (1885) Das Sauerstoff-Bedürfnis des Organismus. Eine farbenanalytische Studie. [On the oxygen consumption of the body. A study using intravital dyes.]. Berlin: Verlag von August Hirschwald.

[4] Lewandowski M (1900) Zur Lehre von der Cerebrospinalflüssigkeit. [On the cerebrospinal fluid.]. Z Klin Forsch 40: 480–494.

[5] Reese TS, Karnovsky MJ (1967) Fine structural localization of a blood–brain barrier to exogenous peroxidase. J Cell Biol 34: 207–217. 6033532 10.1083/jcb.34.1.207 PMC2107213

[6] Wolburg H, Lippoldt A (2002) Tight junctions of the blood-brain barrier: development, composition and regulation. Vascul Pharmacol 38: 323–337. 12529927 10.1016/s1537-1891(02)00200-8

[7] Zlokovic BV (2008) The blood-brain barrier in health and chronic neurodegenerative disorders. Neuron 57: 178–201. 18215617 10.1016/j.neuron.2008.01.003

[8] Liebner S, Fischmann A, Rascher G, Duffner F, Grote EH, et al (2000) Claudin-1 and claudin-5 expression and tight junction morphology are altered in blood vessels of human glioblastoma multiforme. Acta Neuropathol 100: 323–331. 10965803 10.1007/s004010000180

[9] Hawkins BT, Davis TP (2005) The blood-brain barrier/neurovascular unit in health and disease. Pharmacol Rev 57: 173–185. 15914466 10.1124/pr.57.2.4

[10] Stewart PA, Wiley MJ (1981) Developing nervous tissue induces formation of blood-brain barrier characteristics in invading endothelial cells: a study using quail–chick transplantation chimeras. Dev Biol 84: 183–192. 7250491 10.1016/0012-1606(81)90382-1

[11] Arthur FE, Shivers RR, Bowman PD (1987) Astrocyte-mediated induction of tight junctions in brain capillary endothelium: an efficient in vitro model. Brain Res 433: 155–159. 3676853 10.1016/0165-3806(87)90075-7

[12] Janzer RC, Raff MC (1987) Astrocytes induce blood–brain barrier properties in endothelial cells. Nature 325: 253–257. 3543687 10.1038/325253a0

[13] Bell RD, Winkler EA, Singh I, Sagare AP, Deane R, et al (2012) Apolipoprotein E controls cerebrovascular integrity via cyclophilin A. Nature 485: 512–516. 22622580 10.1038/nature11087 PMC4047116

[14] Dente CJ, Steffes CP, Speyer C, Tyburski JG (2001) Pericytes augment the capillary barrier in in vitro cocultures. J Surg Res 97: 85–91. 11319886 10.1006/jsre.2001.6117

[15] Winkler EA, Bell RD, Zlokovic BV (2011) Central nervous system pericytes in health and disease. Nat Neurosci 14: 1398–1405. 22030551 10.1038/nn.2946 PMC4020628

[16] Armulik A, Genove G, Mäe M, Nisancioglu MH, Wallgard E, et al (2010) Pericytes regulate the blood-brain barrier. Nature 468: 557–561. 20944627 10.1038/nature09522

[17] Bell RD, Winkler EA, Sagare AP, Singh I, LaRue B, et al (2010) Pericytes control key neurovascular functions and neuronal phenotype in the adult brain and during brain aging. Neuron 68: 409–427. 21040844 10.1016/j.neuron.2010.09.043 PMC3056408

[18] Daneman R, Zhou L, Kebede AA, Barres BA (2010) Pericytes are required for blood-brain barrier integrity during embryogenesis. Nature 468: 562–566. 20944625 10.1038/nature09513 PMC3241506

[19] Seiffert E, Dreier JP, Ivens S, Bechmann I, Tomkins O, et al (2004) Lasting blood-brain barrier disruption induces epileptic focus in the rat somatosensory cortex. J Neurosci 24: 7829–7836. 15356194 10.1523/JNEUROSCI.1751-04.2004 PMC6729929

[20] Dirnagl U, Iadecola C, Moskowitz MA (1999) Pathobiology of ischaemic stroke: an integrated view. Trends Neurosci 22: 391–397. 10441299 10.1016/s0166-2236(99)01401-0

[21] Mergenthaler P, Dirnagl U, Meisel A (2004) Pathophysiology of stroke: lessons from animal models. Metab Brain Dis 19: 151–167. 15554412 10.1023/b:mebr.0000043966.46964.e6

[22] Endres M, Dirnagl U, Moskowitz MA (2009) The ischemic cascade and mediators of ischemic injury. Handb Clin Neurol 92: 31–34. 18790268 10.1016/S0072-9752(08)01902-7

[23] del Zoppo GJ (2010) The neurovascular unit in the setting of stroke. J Intern Med 267: 156–171. 20175864 10.1111/j.1365-2796.2009.02199.x PMC3001328

[24] Tarawneh R, Galvin JE (2010) Potential future neuroprotective therapies for neurodegenerative disorders and stroke. Clin Geriatr Med 26: 125–147. 20176298 10.1016/j.cger.2009.12.003 PMC2828394

[25] Yang Y, Estrada EY, Thompson JF, Liu W, Rosenberg GA (2007) Matrix metalloproteinase-mediated disruption of tight junction proteins in cerebral vessels is reversed by synthetic matrix metalloproteinase inhibitor in focal ischemia in rat. J Cereb Blood Flow Metab 27: 697–709. 16850029 10.1038/sj.jcbfm.9600375

[26] Bauer AT, Bürgers HF, Rabie T, Marti HH (2010) Matrix metalloproteinase-9 mediates hypoxia-induced vascular leakage in the brain via tight junction rearrangement. J Cereb Blood Flow Metab 30: 837–848. 19997118 10.1038/jcbfm.2009.248 PMC2949161

[27] Mark KS, Davis TP (2002) Cerebral microvascular changes in permeability and tight junctions induced by hypoxia-reoxygenation. Am J Physiol Heart Circ Physiol 282: 1485–1494. 10.1152/ajpheart.00645.2001 PMC3918411 11893586

[28] Jiao H, Wang Z, Liu Y, Wang P, Xue Y (2011) Specific role of tight junction proteins claudin-5, occludin, and ZO-1 of the blood-brain barrier in a focal cerebral ischemic insult. J Mol Neurosci 44: 130–139. 21318404 10.1007/s12031-011-9496-4

[29] Fischer S, Wobben M, Kleinstück J, Renz D, Schaper W (2000) Effect of astroglial cells on hypoxia-induced permeability in PBMEC cells. Am J Physiol Cell Physiol 279: 935–944. 10.1152/ajpcell.2000.279.4.C935 11003573

[30] McCaffrey G, Willis CL, Staatz WD, Nametz N, Quigley CA, et al (2009) Occludin oligomeric assemblies at tight junctions of the blood-brain barrier are altered by hypoxia and reoxygenation stress. J Neurochem 110: 58–71. 19457074 10.1111/j.1471-4159.2009.06113.x PMC3313603

[31] Meairs S, Wahlgren N, Dirnagl U, Lindvall O, Rothwell P, et al (2006) Stroke research priorities for the next decade – A representative view of the European scientific community. Cerebrovasc Dis 22: 75–82. 16790993 10.1159/000093098

[32] Endres M, Engelhardt B, Koistinaho J, Lindvall O, Meairs S, et al (2008) Improving outcome after stroke: overcoming the translational roadblock. Cerebrovasc Dis 25: 268–278. 18292653 10.1159/000118039

[33] Jiang J, Wang W, Sun YJ, Hu M, Li F, et al (2007) Neuroprotective effect of curcumin on focal cerebral ischemic rats by preventing blood-brain barrier damage. Eur J Pharmacol 561: 54–62. 17303117 10.1016/j.ejphar.2006.12.028

[34] Qin Z, Karabiyikoglu M, Hua Y, Silbergleit R, He Y, et al (2007) Hyperbaric oxygen-induced attenuation of hemorrhagic transformation after experimental focal transient cerebral ischemia. Stroke 38: 1362–1367. 17322079 10.1161/01.STR.0000259660.62865.eb

[35] Liu W, Sood R, Chen Q, Sakoglu U, Hendren J, et al (2008) Normobaric hyperoxia inhibits NADPH oxidase-mediated matrix metalloproteinase-9 induction in cerebral microvessels in experimental stroke. J Neurochem 107: 1196–1205. 18786175 10.1111/j.1471-4159.2008.05664.x PMC2582595

[36] Wang G, Guo Q, Hossain M, Fazio V, Zeynalov E, et al (2009) Bone marrow-derived cells are the major source of MMP-9 contributing to blood–brain barrier dysfunction and infarct formation after ischemic stroke in mice. Brain Res 1294: 183–192. 19646426 10.1016/j.brainres.2009.07.070 PMC2758551

[37] Zhao Y, Li Z, Wang R, Wie J, Li G, et al (2010) Angiopoietin 1 counteracts vascular endothelial growth factor-induced blood–brain barrier permeability and alleviates ischemic injury in the early stages of transient focal cerebral ischemia in rats. Neurol Res 32: 748–755. 19660197 10.1179/016164109X12445616596562

[38] Roseth S, Fykse EM, Fonnum F (1995) Uptake of L-glutamate into rat brain synaptic vesicles: effect of inhibitors that bind specifically to the glutamate transporter. J Neurochem 65: 96–103. 7790899 10.1046/j.1471-4159.1995.65010096.x

[39] Roseth S, Fykse EM, Fonnum F (1998) Uptake of L-glutamate into synaptic vesicles: competitive inhibition by dyes with biphenyl and amino- and sulphonic acid-substituted naphthyl groups. Biochem Pharmacol 56: 1243–1249. 9802337 10.1016/s0006-2952(98)00200-7

[40] O'Collins VE, Macleod MR, Donnan GA, Horky LL, van der Worp BH, et al (2006) 1,026 experimental treatments in acute stroke. Ann Neurol 59: 467–477. 16453316 10.1002/ana.20741

[41] Young AR, Ali C, Duretête A, Vivien D (2007) Neuroprotection and stroke: time for a compromise. J Neurochem 103: 1302–1309. 17727635 10.1111/j.1471-4159.2007.04866.x

[42] Fisher M, Feuerstein G, Howells DW, Hurn PD, Kent TA, et al (2009) Update of the stroke therapy academic industry roundtable preclinical recommendations. Stroke 40: 2244–2250. 19246690 10.1161/STROKEAHA.108.541128 PMC2888275

[43] Durukan A, Tatlisumak T (2007) Acute ischemic stroke: overview of major experimental rodent models, pathophysiology, and therapy of focal cerebral ischemia. Pharmacol Biochem Behav 87: 179–197. 17521716 10.1016/j.pbb.2007.04.015

[44] Zhang RL, Chopp M, Zhang ZG, Jiang Q, Ewing JR (1997) A rat model of focal embolic cerebral ischemia. Brain Res 766: 83–92. 9359590 10.1016/s0006-8993(97)00580-5

[45] Menzies SA, Hoff JT, Betz AL (1991) Middle cerebral artery occlusion in rats: a neurological and pathological evaluation of a reproducible model. Neurosurgery 31: 100–106. 10.1227/00006123-199207000-00014 1641086

[46] Michalski D, Grosche J, Pelz J, Schneider D, Weise C, et al (2010) A novel quantification of blood-brain barrier damage and histochemical typing after embolic stroke in rats. Brain Res 1359: 186–200. 20732314 10.1016/j.brainres.2010.08.045

[47] Sixt M, Engelhardt B, Pausch F, Hallmann R, Wendler O, et al (2001) Endothelial cell laminin isoforms, laminins 8 and 10, play decisive roles in T cell recruitment across the blood-brain barrier in experimental autoimmune encephalomyelitis. J Cell Biol 153: 933–946. 11381080 10.1083/jcb.153.5.933 PMC2174323

[48] Ito U, Hakamata Y, Kawakami E, Oyanagi K (2011) Temporary cerebral ischemia results in swollen astrocytic end-feet that compress microvessels and lead to delayed focal cortical infarction. J Cereb Blood Flow Metab 31: 328–338. 20588315 10.1038/jcbfm.2010.97 PMC3049496

[49] Diener HC, Lees KR, Lyden P, Grotta J, Davalos A, et al (2008) NXY-059 for the treatment of acute stroke: pooled analysis of the SAINT I and II Trials. Stroke 39: 1751–1758. 18369171 10.1161/STROKEAHA.107.503334

[50] Bräuninger S, Kleinschnitz C (2009) Rodent models of focal cerebral ischemia: procedural pitfalls and translational problems. Exp Transl Stroke Med 1: 8. 20150986 10.1186/2040-7378-1-8 PMC2820446

[51] Nagaraja TN, Keenan KA, Fenstermacher JD, Knight RA (2008) Acute leakage patterns of fluorescent plasma flow markers after transient focal cerebral ischemia suggest large openings in blood–brain barrier. Microcirculation 15: 1–14. 17934962 10.1080/10739680701409811

[52] Chen B, Friedman B, Cheng Q, Tsai P, Schim E, et al (2009) Severe blood–brain barrier disruption and surrounding tissue injury. Stroke 40: e666–e674. 19893002 10.1161/STROKEAHA.109.551341 PMC2819286

[53] Spirig R, Gajanayake T, Korsgren O, Nilsson B, Rieben R (2008) Low molecular weight dextran sulfate as complement inhibitor and cytoprotectant in solid organ and islet transplantation. Mol Immunol 45: 4084–4094. 18722664 10.1016/j.molimm.2008.07.024

[54] Bechmann I, Galea I, Perry VH (2007) What is the blood-brain barrier (not)? Trends Immunol 28: 5–11. 17140851 10.1016/j.it.2006.11.007

[55] Engelhardt B (2011) β1-integrin/matrix interactions support blood-brain barrier integrity. J Cereb Blood Flow Metab 31: 1969–1971. 21772311 10.1038/jcbfm.2011.98 PMC3208158

[56] Osada T, Gu YH, Kanazawa M, Tsubota Y, Hawkins BT, et al (2011) Interendothelial claudin-5 expression depends on cerebral endothelial cell-matrix adhesion by β(1)-integrins. J Cereb Blood Flow Metab 31: 1972–1985. 21772312 10.1038/jcbfm.2011.99 PMC3208159

[57] Hom J, Dankbaar JW, Soares BP, Schneider T, Cheng SC, et al (2011) Blood-brain barrier permeability assessed by perfusion CT predicts symptomatic hemorrhagic transformation and malignant edema in acute ischemic stroke. AJNR Am J Neuroradiol 32: 41–48. 20947643 10.3174/ajnr.A2244 PMC7964964

[58] Liu J, Jin X, Liu KJ, Liu W (2012) Matrix metalloproteinase-2-mediated occludin degradation and caveolin-1-mediated claudin-5 redistribution contribute to blood-brain barrier damage in early ischemic stroke stage. J Neurosci 32: 3044–3057. 22378877 10.1523/JNEUROSCI.6409-11.2012 PMC3339570

[59] Yu H, Wang P, An P, Yixue X (2012) Recombinant human angiopoietin-1 ameliorates the expressions of ZO-1, occludin, VE-cadherin, and PKCα signaling after focal cerebral ischemia/reperfusion in rats. J Mol Neurosci 46: 236–247. 21710361 10.1007/s12031-011-9584-5

[60] Matsuzaki M, Takahashi R, Nakayama T, Shishikura K, Suzuki H, et al (2010) Disruption of endothelial tight junctions in a patient with mitochondrial encephalomyopathy, lactic acidosis and stroke-like episodes (MELAS). Neuropediatrics 41: 72–74. 20799154 10.1055/s-0030-1261886

[61] Piontek J, Winkler L, Wolburg H, Müller SL, Zuleger N, et al (2008) Formation of tight junction: determinants of homophilic interaction between classic claudins. FASEB J 22: 146–158. 17761522 10.1096/fj.07-8319com

[62] Hamm S, Dehouck B, Kraus J, Wolburg-Buchholz K, Wolburg H, et al (2004) Astrocyte mediated modulation of blood-brain barrier permeability does not correlate with a loss of tight junction proteins from the cellular contacts. Cell Tissue Res 315: 157–166. 14615934 10.1007/s00441-003-0825-y

[63] Ito U, Ohno K, Yamaguchi T, Takei H, Tomita H, et al (1980) Effect of hypertension on blood-brain barrier. Change after restoration of blood flow in post-ischemic gerbil brains. An electronmicroscopic study. Stroke 11: 606–611. 7210066 10.1161/01.str.11.6.606

[64] Dietrich WD, Busto R, Watson BD, Scheinberg P, Ginsberg MD (1987) Photochemically induced cerebral infarction. II. Edema and blood-brain barrier disruption. Acta Neuropathol 72: 326–334. 3577688 10.1007/BF00687263

[65] Wang Y, Roche O, Xu C, Moriyama EH, Heir P, et al (2012) Hypoxia promotes ligand-independent EGF signalling via hypoxia-inducible factor-mediated upregulation of caveolin-1. Proc Natl Acad Sci USA 109: 4892–4897. 22411794 10.1073/pnas.1112129109 PMC3323978

[66] Belayev L, Busto R, Zhao W, Ginsberg MD (1996) Quantitative evaluation of blood-brain barrier permeability following middle cerebral artery occlusion in rats. Brain Res 739: 88–96. 8955928 10.1016/s0006-8993(96)00815-3

[67] Strbian D, Durukan A, Pitkonen M, Marinkovic I, Tatlisumak E, et al (2008) The blood-brain barrier is continuously open for several weeks following transient focal cerebral ischemia. Neuroscience 153: 175–181. 18367342 10.1016/j.neuroscience.2008.02.012

